# Communication and interaction between native-speaker Norwegian nurses and nurses with Norwegian as a second language in home-based care settings: a qualitative exploratory interpretive study

**DOI:** 10.1186/s12912-026-04834-2

**Published:** 2026-05-30

**Authors:** Ellen Benestad Moi, Anne Valen Skisland, Berit Johannessen, Kristin Haraldstad, Gudrun Rohde, Sylvi Flateland

**Affiliations:** Faculty of Health and Sport Sciences, University of Agder, Post box 422, Kristiansand, 4604 Norway

**Keywords:** Cross-cultural communication, Patient safety, Small talk, Language barriers

## Abstract

**Introduction:**

Language barriers between healthcare personnel can influence patient safety and result in inadequate follow-up and communication of the patient’s needs.

**Aim:**

The aim of this study was to explore how nurses with Norwegian as a first language (NFL) and nurses with Norwegian as a second language (NSL) experience communication and interaction with each other in home-based workplace settings.

**Method:**

The study involved in-depth interviews with 14 nurses: seven nurses with NFL and seven with NSL, and Braun and Clarke’s framework of reflexive thematic analysis were used.

**Results:**

Two main themes were identified: (1) the importance of mutual understanding in professional settings, with subthemes highlighting the need to double-check written communication and clarify oral exchanges; and (2) the value of social interactions among colleagues, emphasizing challenges posed by diverse cultural backgrounds and the necessity for clarification due to different native languages.

**Conclusion:**

This study underscores the communication challenges in interactions between nurses with NFL and NSL in home-based care. Misunderstandings related to limited language skills may pose a notable threat to patient safety. However, despite the language barriers, nurses with NFL and NSL show strong commitment to clarity and quality, and to working in a positive psychosocial work environment. These findings emphasize the need for organizational support, such as intercultural competence training and clear communication routines, to enhance collaboration and patient safety.

**Supplementary Information:**

The online version contains supplementary material available at 10.1186/s12912-026-04834-2.

## Background

From a patient safety perspective, inadequate communication between healthcare workers during the exchange of patient information is a well-documented cause of adverse events and can lead to patient harm [1, 2]. Consequently, effective communication is widely recognized as a cornerstone of safe and high-quality care. However, communication in healthcare does not occur in a vacuum; it is shaped by linguistic proficiency, cultural background, and workplace context [3–5]. Differences in native language and cultural background between healthcare professionals (HCPs) can influence how patient information is interpreted, shared, and acted upon and may pose a threat to patient safety [6–9]. Increasing globalization has greatly increased the ethnic diversity within the healthcare workforce. This shift reflects broader demographic changes in Norwegian society as immigration continues to contribute to workforce diversity. In Norway, immigrants account for over 17% of municipal care workers [10], and the healthcare service sector employs the highest proportion of people with an immigrant background. These immigrants, for whom Norwegian is a second or third language, originate primarily from Asia, followed by Eastern Europe and then Africa [11].

Culturally and linguistically diverse nurses are important within healthcare systems because they provide additional human resources by bringing their diverse experiences and expertise, which adds to the organizational cultural capital [12]. Many immigrants coming to Norway will work in home-based care, given the expansion of this sector because of an aging population and the demand for HCPs. Home-based care in Norway includes various services, of which practical assistance and health services in the home are the most comprehensive. These patients live either in their own private home or in municipally managed housing [13]. Nurses employed in home-based care mainly work independently and meet each other primarily only for handover reports, meal breaks, or when assistance is needed during care situations.

In home-based care, where nurses work largely independently and have limited opportunities for face-to-face interaction, communication relies on brief and often informal exchanges. In this context, linguistic and cultural diversity may affect how confidently, and clearly patient-relevant information is shared, with potential consequences for patient safety [14]. Research indicates that nurses for whom Norwegian is a second language (NSL) may experience challenges relating to language barriers and overcoming feelings of estrangement. However, they also find meaning by being a member of a team and overcoming challenges, such as the need for team interdependency, while appreciating new cultural experiences and striving to belong [15]. The perception of competence among multicultural staff members is intricately linked to educational, ethnic, linguistic, and social dimensions [16, 17].

Previous studies have shown that although the language competence of HCPs with Norwegian as a second language (NSL) is sufficient for performing care-related tasks [7], this competence is largely attributed to the implementation of strict language requirements for staff, which may mitigate some linguistic challenges. Nevertheless, even under such requirements, HCPs with NSL have been reported to be more reluctant to initiate conversations with patients’ relatives, indicating that communication challenges may persist beyond formal language proficiency [7].

At the same time, research examining the benefits of a multicultural workforce suggests that diversity can contribute to patient safety and higher-quality care services [12, 18]. However, nurses using a second language frequently encounter communication challenges when collaborating with majority-language-speaking colleagues [18–22]. The aim of such cross-cultural communication in healthcare is mutual understanding between individuals from different cultural backgrounds, including interactions between insiders (such as family and friends) and outsiders, including HCPs [23]. Such communication involves both verbal and nonverbal elements, as well as the patient’s health literacy, and may be complicated by differing perspectives and interpretations. Consequently, differences in communication, education, values, cultural expectations, power relations, and experiences of social avoidance or rejection represent key challenges for nurses with an immigrant background adapting to the Norwegian clinical context [24].

Cross-cultural communication can create challenges in communication between nurses, particularly in home-based care settings where informal and direct communication plays a vital role in service delivery. Home care nurses communicate with other HCPs primarily via telephone, secure messaging, email or brief verbal report exchanges. Globally, patient safety is key area of concern, as emphasized in the World Health Organization’s Global Patient Safety Action Plan for 2021–2030, which highlights the importance of effective and robust communication systems and intercultural competence in the health services [4, 25, 26]. Despite this emphasis, research examining communication dynamics between nurses with Norwegian as a first language (NFL) and those with NSL in Norway’s home-based care settings remains limited. Communication and interactions include both professional and informal communication. While minority HCPs experiences have been explored in various contexts, there is insufficient knowledge of how everyday communication and interaction between nurses with different linguistic and cultural backgrounds may influence patient safety, the working environment, the organizational culture and psychological safety in home-based care. Communication barriers may hinder information exchange, discourage speaking up, and contribute to social exclusion, with potential consequences for both care quality and staff wellbeing.

The aim of this study was to explore how nurses with NFL and NSL experience communication and interactions with each other in a home-based workplace setting, with particular attention to implications for patient safety, the working environment, and psychological safety.

## Methods

### Design

Our qualitative study used an exploratory, interpretive design to capture the experiences of nurses with NFL and NSL regarding their communication and interactions with each other in Norway’s home-based care settings. 

### Study setting and recruitment 

Home care nurses employed in the community health services in Southern Norway were recruited for individual interviews between February and April 2024. The participants were selected using strategic sampling. A senior manager initially contacted local managers within the municipality (approximately 175,000 inhabitants) and asked them to assist with recruitment by approaching their staff and inviting nurses to participate in the interview study. Local managers distributed study information to potential participants, and those who expressed interest contacted the researcher directly. The recruited nurses were employed in different districts within the municipality. Our objective was to include an equal number of nurses with prior experience in communication and collaboration with nurses with NSL; our aim was to include half of the participants with NSL and the other half with NFL. Nurses interested in participating were then contacted by one of the authors, and interviews were scheduled at times and locations convenient for the participants. A total of 14 nurses were interviewed: seven nurses with NFL and seven with NSL.

### Inclusion and exclusion criteria

To be eligible for inclusion the nurses had to be working part or full time as a home care nurse in the community health services and using Norwegian as their first or second language. The exclusion criteria were being unable to understand or express themselves in the Norwegian language, which would limit their ability to provide informed consent or undertake an in-depth interview.

The age of the nurses ranged from 21 to 61 years (median = 33 years), and they had worked from 1 to 19 years (median = 4.5 years) as a home care nurse in community health. The sample included 10 female and four male nurses. The nurses with NSL originated from Asia, Africa, and Western Europe, and had been in Norway from 8 to 35 years (median = 14 years) (See Table 1).

**Table 1:** Characteristics of participants

Participant number	Gender	First language	Years living in Norway	Years working in home-based care
1	Man	Norwegian	25 years	1 year
2	Woman	Norwegian	42 years	10 years
3	Man	Norwegian	31 years	6 years
4	Woman	Norwegian	22 years	1 year
5	Woman	Norwegian	27 years	5 years
6	Woman	Norwegian	30 years	7 years
7	Woman	Norwegian	31 years	3 years
8	Woman	Chinese	27 years	1 year
9	Woman	Arabic	15 years	2 years
10	Woman	African	14 years	1 year
11	Man	African	12 years	5 years
12	Woman	French	35 years	19 years
13	Man	Spanish	8 years	8 years
14	Woman	Spanish	8 years	4 years

### Data generation

We developed a semi-structured interview guide and asked the nurses questions such as, “How do you experience the communication with your colleagues?”; “What, if any, challenges exist regarding language? Please provide examples if possible”; and “How do different languages and cultural backgrounds impact your work life?” The interview guide also included potential probes for exploring key aspects for achieving deep and rich descriptions. This provided a flexible and exploratory approach that enabled the interviewer to stay on topic while still responding to information the nurses shared or discussed.

The authors (EMB, BJ, and SF) conducted the interviews in person, in the nurse’s workplace or at a place convenient for the nurse (*n* = 12) or digitally using Zoom Meetings (*n* = 2). All three interviewers have experience as nurses across different clinical fields as well as substantial expertise in qualitative research; however, at the time of data collection, they were all employed as university staff teaching in bachelor’s and master’s programmes in nursing. The interviews began with an introduction of the interviewer and an explanation of their interest in the research topic and lasted between 18 and 37 min (median = 30 min). No repeat interviews were conducted. Each interview was recorded digitally and subsequently transcribed. After each session, the interviewers wrote brief notes to capture their initial thoughts and to facilitate reflection on their role in the data construction process.

### Data analysis

A reflexive thematic analysis (Braun & Clarke) was conducted within an interpretive–constructivist epistemological framework, recognising the active role of the researcher in the construction of themes [27]. They describe a process comprising six phases: (1) Familiarization with the dataset, (2) Coding, (3) Generating initial themes, (4) Developing and reviewing themes, (5) Refining, defining and naming themes, and (6) Writing up [27].

Three authors (SF, AVS, and EBM) were main responsible for the initial analyses, involving a thorough and iterative review of the transcripts to identify potential codes. These codes were then systematically compared, refined, and organized into subthemes and overarching themes. Subsequently, all authors, all of whom are registered nurses with solid experience in qualitative research, engaged in prolonged and collaborative analytical discussions to agree upon, refine, define, and name these themes and subthemes. This iterative process facilitated a transition from predominantly semantic to more latent-level themes (See Table 2).

**Table 2:** Examples of the qualitative thematic analysis process

Example of text coded	Codes	Subthemes	Main themes
“I always ask the person next to me if it’s okay to write like this”	Seeking validation	Double-checking of written communication	The significance of understanding and being understood in a professional setting
“I have the words in my head in my own language”	Thoughts are native	Increased need for clarification in oral communication	
“I am a very social person who likes to talk a lot and share experiences. In my culture, we might share too much. Yes, but I am used to sharing. Sitting at a table and not being able to share… sometimes one can feel a bit frustrated… I become much, much quieter than I usually am”	Becoming quieter than usual	Challenges of different cultural and social backgrounds	The importance of social interactions with colleagues
“… details from social conversations during breaks due to fast talking and lots of laughter – I don’t catch the points. When it’s brought up, colleagues who are native Norwegian speakers say, ‘Oh, but that’s OK, you understand everything we say.’ That is not true. Sometimes it’s a bit difficult to keep up in that regard. It’s challenging to share stories because of language barriers, speed, and quick shifts in focus.”	Missing informal communication	Different native languages create a need for clarification	

## Results

In the thematic analysis the researchers constructed two overarching main theme: 1) the significance of understanding and being understood in a professional setting with subthemes (a) double-checking of written communication and (b) increased need for clarification in oral communication, and main theme 2) the importance of social interaction with colleagues with subthemes (a) the challenge of different cultural and social backgrounds and (b) different native languages create a need for clarification.

### Significance of understanding and being understood in a professional setting

The level of Norwegian language proficiency was described as highly variable. Some nurses with NSL spoke Norwegian very well, especially those who had completed their education in Norway. All informants noted that additional clarification was needed in both written and oral communication, which increased the workload and the time needed to ensure the accuracy of information.

#### Double-checking of written documentation

Written documentation and reports were challenging, especially for nurses with NSL (Participants 1, 2, 3, 4, 5, 6 and 9). Several of all the informants noted that nurses with NSL often wanted their colleagues with NFL to review their written documentation as they found it difficult to be precise in Norwegian. One nurse with NSL said, “*I have the words in my head in my own language*” (Participant 9). Another nurse noted that it could be random who was asked to review written documentation, stating, “*I always ask the person next to me if it’s okay to write like this*” (Participant 12). The need for confirmation of written documentation was expressed by both nurses with NFL and NSL, particularly when documents were intended for external or public institutions.

Reading and comprehending written documentation were also highlighted as a challenge for nurses with NSL because they required more time to read and comprehend the specific details of reports. Despite this, not all nurses with NSL were able to understand exactly what they were expected to do at the patient’s home when reviewing reports. One nurse with NSL commented, “*When I read the report*,* it’s not easy to understand the details*,* but I grasp the overall picture*” (Participant 1). Additional time and effort from NFL nurses were used to ensure accurate comprehension. Some nurses reflected that such difficulties may potentially influence the quality of care and, in certain situations, may potentially have implications for patient safety if critical details are overlooked or misinterpreted.

One of the NFL nurses mentioned that it could be difficult for some nurses with NSL to understand which names were male or female when reading the patient records. Moreover, they struggled to understand words and expressions about the patient’s needs, which led to uncertainty about the patient’s situation (Participant 3).

#### Increased need for clarification in oral communication

Nuances in language could create significant challenges between NSL and NFL nurses, especially when messages needed to be conveyed over the phone instead of in person (Participants 3, 9 and 10). All nurses felt a shared need for clearer communication when discussing patient issues with each other. One nurse with NFL commented, “*I feel calmer doing it myself or talking to someone who I know can repeat it exactly … and I almost never do it over the phone*” (Participant 10). Nurses felt it was essential to be aware of the need to use simple words and concepts, and to be as precise as possible in communication. Misunderstandings were common: *”Sometimes they don’t understand whether I’m referring to a single multidose or just a pill organizer. They might return and simply report that the patient had medication*” (Participant 3).

Nurses felt it necessary to conduct oral checks to prevent misunderstandings and ensure effective communication between nurses. This ensures that information regarding the patient’s name, medications, messages, and other details was received accurately and understood. Some nurses felt that those with limited proficiency in the Norwegian language often responded with positive affirmations to statements they did not fully understand; for example, *“They say ‘yes’ because they are afraid to say that they don’t understand…* (Participant 6). Most of the nurses with NSL reported that they often lacked the precise words and terminology needed to convey accurate observations they had made of patients. They used digital language tools to enhance the precision of their verbal expressions.

Misunderstandings because of limited language skills were seen as a significant danger to patient safety, and examples were provided of tasks that were supposed to be completed. Although the person appeared to understand the message, it became evident that they had not and this posed a significant risk to the patient, as noted by one nurse: “*And then*,* if there are things that should have been done*,* maybe they won’t be done*” (Participant 6). Situations in which nurses with NSL have limited Norwegian language skills can also hinder cooperation at all organizational levels; for example, if critical information given or received from relatives has been misunderstood, this may require additional time for follow-up and clarification and potentially compromising patient safety.

The use of digital platforms such as Google Translate was considered useful and safe for ensuring that what they had said was understood. All NSL nurses interviewed used Google Translate as an aid. In some situations, the communication tool “Closed Loop” was used to ensure that information was understood correctly (Participant 3).

Situations were described in which NSL nurses reported that a patient was experiencing pain, but due to language barriers, they struggled to convey whether it was a recent onset or a longstanding condition, making it challenging for NFL nurses to accurately assess and rely on the communicated observations. To ensure professionally responsible healthcare, critical points emerged from the interviews. In certain situations, there was a tendency to receive “*fragments of information from healthcare workers who may not understand the nuances when describing the patient’s situation*” (Participant 3). Additionally, procedures were often presented both orally and in writing, as one NFL noted: “*I don’t just use language but show practically what to do*” (Participant 6).

### Importance of social interaction with colleagues

Navigating workplace relationships seemed to require an understanding of colleagues’ diverse cultural and social backgrounds. Nurses with NSL face communication challenges related to different cultural norms and the use of varied Norwegian dialects. These challenges were described as sometimes leading to perceptions of nurses with NSL as quiet or reserved. Participants reported difficulties in understanding dialects, engaging in informal conversations, and following rapid topic shifts often necessitate clarification in everyday interactions. Despite these challenges, both nurses with NSL and NFL value the interactions that allow sharing of their cultural experiences, which helps to foster a richer, multicultural dialogue within their team and environment.

#### Challenges of different cultural and social backgrounds

The diverse personalities, cultural backgrounds, and social contexts present challenges to the relationships between colleagues. Communication and emotional expression vary significantly between cultures.

Some nurses with NSL from an Asian or African background described nurses with NFL as very direct and having the expectation of looking the other person in the eye during conversations (Participants 8, 11, 12 and 13). In some African cultures, it is rude to look the other person in the eye while talking, as noted by a nurse with NSL: “*In my culture*,* I am not used to direct eye contact; it is considered impolite*” (Participant 11). Some nurses with NSL found these expectations demanding, though most did not report shyness during eye contact. Avoiding eye contact was associated with perceptions of shyness and quietness by both NSL and NFL nurses.

Traditions from childhood together with a lack of common experiences from activities in leisure time or time outside of work can also affect relationships because some common reference points may be missing. Some NFL nurses noted that nurses from an African background may talk loudly and interrupt each other to get their opinions across (Participants 9 and 12). This was perceived as possibly rude and unfamiliar by nurses with NFL. Some of the nurses with NSL struggled to understand what was said in social settings with colleagues (Participants 8, 9, 10 and 11). They reflected about whether it was related to culture or a lack of language comprehension that caused misunderstandings. Some nurses with NFL frequently responded with slightly embarrassed laughter to much of what was being said. This behaviour could be interpreted as an attempt to conceal a lack of understanding, a need to mask vulnerability, or simply as an expression of their inherent personality traits.

A lack of shared experiences from childhood, leisure activities, spending time with friends and family, and traditions for various holidays also challenged the relationships between colleagues. Two nurses with NSL felt left out when the weekend’s unfamiliar activities were referred to during breaks, or places that were unknown were discussed (Participants 8 and 10). It was the unknown or unfamiliar and the lack of reference points that presented challenges in their interactions with others. At the same time, being with others during breaks provided a greater understanding of culture and social codes, which was applied further socially and in interactions with patients. A nurse with NSL noted that colleagues with NSL from the same country and cultural background shared a unique bond that was lacking for this individual who was the sole representative from their country in their workplace (Participant 14). Additionally, this participant’s nursing education was completed outside of Norway.

Despite these challenges, all nurses felt respected and included in the work environment, regardless of their cultural and social background. Nurses with NSL found it positive to be asked about their own background, upbringing, and views on women, food, and traditions (Participants 8, 9, 10, 11 and 13). Nurses with NFL found it interesting to hear about the different cultures of their colleagues, although no nurses with NFL said they spoke or were asked about their own traditions.

#### Need for clarification because of different native languages

The presence of diverse native languages introduced challenges and led to misunderstandings in communication and interactions between HCPs, which sometimes caused frustration and a sense of inadequacy in some contexts. Almost all nurses with NSL said that nuances in the language, especially different Norwegian dialects and numbers, were difficult to understand.

Although nurses with NSL did not find professional language challenging, they struggled with casual small talk in Norwegian given that they were not accustomed to such topics (Participants 8, 9, 10 and 11). Consequently, some nurses with NSL found it challenging to share personal and everyday experiences, often requiring clarifying questions to effectively understand and participate in small talk. One nurse with NFL presumed that nurses from an immigrant background found everyday language easier to communicate than professional language. Additionally, maintaining the flow of conversation was challenging because nurses with NFL speak quickly and topics can shift frequently. In contrast, some nurses with NFL noted that some nurses with NSL also speak quickly, and that comprehension did not appear to be hindered. Neverless, nurses with NSL seemed to concentrate more and often asked clarifying questions.

All nurses mentioned misunderstandings when dealing with numbers, place names, and gender-specific names. These issues arose in both social interactions and during the exchange of patient information. Misunderstandings forced nurses with NFL to modify their different Norwegian dialects and specify whether names referred to places or individuals (Participants 1, 3, 4 and 7). Nonetheless, some nurses with NSL considered such situations crucial for learning everyday Norwegian, whereas other nurses with NSL found humour in misunderstandings related to name–gender confusions: *“Or sometimes it can be a little funny when I pronounce or interpret the gender of the first name wrong […]*,*”* (Participant 11).

Nurses with NSL reported positive experiences in situations in which they were able to convey aspects of their true selves on a personal level. These successful interactions typically occurred in the presence of only two or three colleagues. On the other hand, some nurses with NSL felt frustrated because they had trouble expressing their thoughts because of their limited vocabulary. This language barrier made them unsure, and they worried about choosing the right words, which led them to feel somewhat discouraged, as they were used to sharing, and wanted to share, more about their personal lives. This situation also led them to be quieter than normal, which was a challenge to expressing their true self: “I can’t express myself very well, can’t explain as you want […], it can be frustrating […].” (Participant 14).

## Discussion

This study explored how nurses with NFL and NSL experience communication and interactions with each other in a home-based workplace setting. Home-based care is unique in that nurses frequently engage in everyday, unscheduled communication with patients and relatives, where linguistic nuances and cultural expectations may significantly influence information accuracy and patient safety. In addition, limited shared meeting points with other healthcare personnel increase nurses’ reliance on clear, precise communication to prevent misunderstandings that could compromise care. The findings revealed that language barriers and cultural differences challenged both professional and social interactions, often requiring additional time to ensure safe and high-quality care.

The interview results indicate that collaboration between NSL and NFL nurses often leads to uncertainty in understanding written and verbal communication. NSL nurses were concerned about the burden of ensuring accuracy in their written communication. Similarly, NFL nurses needed their documents checked to maintain patient safety and high documentation quality, emphasizing shared dedication to thorough quality assurance.

This aligns with research suggesting that streamlining health services without an impact assessment can compromise nursing service quality and patient safety [28]. Effective changes to nursing handover reports require cultural, role, and behaviour shifts, along with tailored approaches, time, education, and support [29].

Both nurses with NFL and NSL noted the need for clarification in oral communication. They used various digital translation platforms and communication tools, including closed loop communication. Closed loop communication is a well-established tool designed to ensure a shared understanding of situations, which in turn helps to maintain quality and patient safety [30]. However, the quality of the different digital translation platforms used may vary and this issue should be examined.

Misunderstandings related to numbers, place names, gender-specific names, and dialects were often seen as amusing and, after clarification, this attitude reflected a good atmosphere within the workplace. Such misunderstandings did not cause nurses with NSL to feel insecure. This finding supports the description of Egede-Nissen, Sellevold [15] that experiences of alienation are alleviated as nurses with NSL adapt to the new culture by sharing more about themselves. However, the findings contrast with those of Debesay, Arora [7], who claim that nurses with NSL experience exclusion and marginalization.

In our study, the use of humour in situations involving misunderstandings appears to signal a psychologically safe work environment, where nurses felt comfortable asking questions, clarifying information, and speaking up without fear of embarrassment or rejection. Psychological safety is a key factor for effective teamwork and patient safety, particularly in home-based care, where nurses work independently and rely on brief and often remote communication [31]. In such settings, organizational culture and leadership play an important role in enabling open communication that promote patient safety [32, 33].

In contrast to previous research [7, 12, 34, 35], we found no evidence in the interviews suggestive of irritation or negativity towards colleagues with NSL. This raises the question of whether the nurses with NFL in our study avoided problematizing or addressing this issue, possibly because of the small sample of NSL nurses or concerns about appearing discriminatory.

Although there are few common meeting points during a shift for home care nurses, all nurses in our study felt respected and included in the social work community, regardless of their cultural background. This does not align with previous research that has reported that nurses with NSL struggle to experience a sense of belonging and feel discriminated against because of their different cultures, beliefs, and values [12, 15]. Feeling respected and experiencing belonging are important factors in interpersonal interactions and are integral to creating a safe psychosocial work environment [19, 31].

The participants in this study described situations that highlighted differing understandings of social norms. In this context, social norms are understood as informal rules within a group or society that prescribe or prohibit behaviours in which members of the group may engage [36]. Both nurses with NFL and NSL described how behaviours that are accepted and common within Norwegian culture could lead to misunderstandings. For instance, some nurses with NSL did not maintain eye contact during communication or interrupted conversations. Some nurses with NSL laughed at or answered “yes” to everything being said and were perceived as not understanding the content of the communication, which aligns with other findings. Egede-Nissen, Sellevold [15] reported that nurses with NSL “conceal their lack of understanding” and Purnell [23] noted that, in some cultures, “yes” does not necessarily mean agreement with what is being asked or being explained but rather “I hear you.” To avoid such misunderstandings, nurses need to exercise caution in framing questions and interpreting responses to yes/no questions [23].

NSL nurses reported challenges in discussing leisure activities due to linguistic and cultural differences, recognizing the need to share more personal information. Difficulties in finding the right words often led to misunderstandings, resulting in uncertainty and sometimes silence during informal conversations. This is consistent with previous research showing that informal conversations are important in the psychological work environment [19, 31]. It is important to recognize that norms for personal disclosure vary across cultures; in some cultures, sharing private information about oneself or one’s family with colleagues is common [23]. Achieving mutual understanding in such contexts may therefore require colleagues or managers to ask clarifying questions and actively facilitate inclusive communication to support a healthy work environment.

In this context, we note that one nurse with NFL in our study assumed that nurses with NSL found everyday language easier to communicate than professional language. This highlights a possible mismatch that needs to be addressed clearly in the work environment.

When NSL nurses in our study work in a new and unfamiliar culture, they need to develop cultural competence. Previous research shows that this involves receiving explanations, maintaining openness, and stepping outside one’s comfort zone [37]. Our findings indicate that NSL nurses actively strive to develop the ability and readiness to work effectively within the patient’s cultural context, which aligns with Campinha-Bacote’s model of cultural competence [38].

Communication is crucial when nurses from different cultures work together, both in clinical practice and in the social interactions of the workplace. The intercultural communication competence model by Dai and Chen [39] provides a relevant framework, describing competence as comprising knowledge (cognitive), motivation (affective), and skills (behavioral). Developing these dimensions appears essential for managing cross-cultural challenges related to language, cultural, and emotional aspects of care.

All nurses reported a psychologically safe work environment, where they felt comfortable asking questions and seeking clarification. Despite linguistic and cultural differences, the environment was described as respectful and inclusive, reflecting a strong commitment to safe and high-quality care. A psychosocial work environment encompasses both psychological factors, related to how work demands and roles are experienced, and social factors concerning interpersonal relationships and communication in the workplace [31, 33, 40]. Previous research has shown that challenges in the psychosocial work environment are often linked to unclear organizational structures and insufficient leadership support, which may contribute to uncertainty, reduced reflection, and strained collaboration among staff [14].

Psychological safety has been identified as a key mechanism through which organizational culture and leadership influence communication, teamwork, and patient safety. Supportive leadership that encourages openness and reflection enables healthcare professionals to speak up and seek clarification, whereas limited leadership engagement may negatively affect staff well-being and patient safety [41, 42].

The findings emphasize the need for organizational policies that support effective communication and the professional integration of migrant nurses. This is particularly relevant in Norwegian municipal healthcare services, where assistant nurses and unskilled workers constitute the majority of the workforce and where the proportion of employees with an immigrant background is substantially higher than among registered nurses.

By illuminating everyday communication and interaction between nurses with NFL and NSL in home-based care settings, this study contributes new knowledge to an underexplored area. The findings extend existing research beyond formal language proficiency to include informal and relational dimensions of workplace communication, highlighting their significance for patient safety and psychological safety.

### Strengths and limitations

A notable strength of our study is that the group of nurses came from broad and varied backgrounds in terms of age, gender, years of experience, and workplaces, which provided different perspectives of knowledge and organizational structures. The varied backgrounds and the richness of the data they provided enabled us to identify the contextualized descriptions of their distinct voices. This diversity enhances the credibility and depth of the findings by incorporating a broad spectrum of perspectives on organizational structures and interprofessional dynamics. None of the participants dropped out of the study. Another strength is that the study was conducted by highly knowledgeable researchers who, collectively, brought their methodological expertise and contextual understanding to the project. The Consolidated Criteria for Reporting Qualitative Research [43] were followed, thereby enhancing the transparency of the reporting. Our detailed reporting of the participant characteristics, research context, process of analysis and use of illustrative quotes support the rigour, transparency, and trustworthiness of the study [44]. The authors’ reflexive journaling during data generation and analysis enhanced understanding, interpretation, and reflexivity.

Nevertheless, the study has some limitations. Participants were recruited from workplaces within a limited geographical area in Southern Norway, which may have reduced variation in experiences related to language use, cultural integration, and workplace norms. Although the study is situated within a nationally regulated healthcare system with relatively uniform organisational structures, population characteristics vary, and transferability may therefore be greater to settings with similar demographic and organisational features. In addition, participants may have been particularly motivated to discuss communication, and this self-selection bias may have limited the breadth of perspectives captured and thereby affected transferability.

## Conclusions

This study highlights the challenges experienced in communication between nurses with NFL and NSL in home-based care.

Although language barriers and cultural differences can lead to misunderstandings and may impact patient safety, both nurses with NFL and NSL showed a strong commitment to ensuring clarity and quality in their work. Despite these challenges, the nurses reported a respectful and inclusive work environment.

The findings emphasize the importance of organizational support, including intercultural competence training and clear communication routines, to strengthen collaboration, ensure patient safety, and promote a positive psychosocial work environment in an increasingly multicultural healthcare sector. Most healthcare personnel in the Norwegian Municipal Health Care Services are assistant nurses and unskilled workers, and the proportion of those with an immigrant background is much higher than that among registered nurses. Future research should therefore also focus on this group of employees. It would also be of interest to interview the patients and their relatives about their experiences with health personnel with NSL.

## Supplementary Information

Below is the link to the electronic supplementary material.

Supplementary Material 1

## Data Availability

All data generated or analysed during this study are included in this published article.

## Abbreviations

HCPs: Healthcare professionals
NFL: Norwegian as a first language
NSL: Norwegian as a second language

## References

[1] Patient Safety Advisory Group. Patient safety Global: WHO. 2023 [Available from: https://www.who.int/news-room/fact-sheets/detail/patient-safety

[2] The Joint Comission. Inadequate hand-off communication, Sentinel Event Alert: The Joint Commission, Department of Corporate Communications., 2017 [Available from: https://www.jointcommission.org/-/media/tjc/newsletters/sea-58-hand-off-comm-9-6-17-final2.pdf

[3] Grailey KE, Murray E, Reader T, Brett SJ. The presence and potential impact of psychological safety in the healthcare setting: an evidence synthesis. BMC Health Serv Res. 2021;21(1):773. 34353319 10.1186/s12913-021-06740-6 PMC8344175

[4] Murray JSJ, Gale B, Mossburg S. Ensuring patient and workforce safety culture in healthcare rockville (MD): agency for healthcare research and quality,: US Department of Health and Human Services. 2024. Available from: https://psnet.ahrq.gov/perspective/ensuring-patient-and-workforce-safety-culture-healthcare.

[5] Kumar S. Psychological Safety: What It Is, Why Teams Need It, and How to Make It Flourish. Chest. 2024;165(4):942–9. 37977265 10.1016/j.chest.2023.11.016

[6] Meuter RFI, Gallois C, Segalowitz NS, Ryder AG, Hocking J. Overcoming language barriers in healthcare: A protocol for investigating safe and effective communication when patients or clinicians use a second language. BMC Health Serv Res. 2015;15(1):371. 26357948 10.1186/s12913-015-1024-8 PMC4566365

[7] Debesay J, Arora S, Fougner M. Organisational culture and ethnic diversity in nursing homes: a qualitative study of healthcare workers’ and ward nurses’ experiences. 2022. 10.1186/s12913-022-08184-y PMC9248104 35773681

[8] Brownie S, Chalmers L. English-Only Policies and Allegations of Racism in Nursing: Safety, Culture and Respect Prevail. J Adv Nurs. 2025;81(6):3362–75. 39943781 10.1111/jan.16813 PMC12080073

[9] Skjeggestad E, Gerwing J, Gulbrandsen P. Language barriers and professional identity: A qualitative interview study of newly employed international medical doctors and Norwegian colleagues. Patient Educ Couns. 2017;100(8):1466–72. 28283216 10.1016/j.pec.2017.03.007

[10] Statistics Norway. Immigrants and Norwegian-born to immigrant parents,: Statistics Norway. 2025 [Available from: https://www.ssb.no/en/befolkning/innvandrere/statistikk/innvandrere-og-norskfodte-med-innvandrerforeldre

[11] Statistics Norway. Innvandrerne sto for 1 av 6 årsverk innen omsorg: Statistics Norway; 2018 [Available from: https://www.ssb.no/helse/artikler-og-publikasjoner/innvandrerne-sto-for-1-av-6-arsverk-innen-omsorg

[12] Kamau S, Koskenranta M, Kuivila H, Oikarainen A, Tomietto M, Juntunen J, et al. Integration strategies and models to support transition and adaptation of culturally and linguistically diverse nursing staff into healthcare environments: An umbrella review. Int J Nurs Stud. 2022;136:104377. 36327682 10.1016/j.ijnurstu.2022.104377

[13] Hoen BT, Abrahamsen DR. Sykehjem og hjemmetjenesten i Norge Statistics Norway,2025 [Available from: https://www.ssb.no/helse/helsetjenester/artikler/sykehjem-og-hjemmetjenesten-i-norge.

[14] Schnipper JL FE, Hall KK, et al. US Department of Health and Human Services. Approach to Improving Patient Safety: Communication Rockville (MD): Agency for Healthcare Research and Quality,.: AHRQ; 2021 [Available from: https://psnet.ahrq.gov/perspective/approach-improving-patient-safety-communication.

[15] Egede-Nissen V, Sellevold GS, Jakobsen R, Sørlie V. Minority healthcare providers experience challenges, trust, and interdependency in a multicultural team. Nurs Ethics. 2019;26(5):1326–36. 29575974 10.1177/0969733017752546

[16] Munkejord MC, Tingvold L. Staff perceptions of competence in a multicultural nursing home in Norway. Soc Sci Med. 2019;232:230–7. 31103966 10.1016/j.socscimed.2019.04.023

[17] Christensen K. Life Trajectories of Migrant Care Workers in the Long-Term Care Sectors in Norway and the UK. Social Policy Soc. 2017;16(4):635–44.

[18] Phillips JM, Malone B. Increasing Racial/Ethnic Diversity in Nursing to Reduce Health Disparities and Achieve Health Equity. Public Health Rep. 2014;129(1suppl2):45–50. 24385664 10.1177/00333549141291S209 PMC3863700

[19] Isakov T, Kamau S, Koskenranta M, Kuivila H, Oikarainen A, Ropponen P, et al. Culturally and linguistically diverse nurses’ experiences of how competence facilitates integration into the working environment: A qualitative study. Nurse Educ Pract. 2023;67:103553. 36657318 10.1016/j.nepr.2023.103553

[20] Brunton M, Cook C. Dis/Integrating cultural difference in practice and communication: A qualitative study of host and migrant Registered Nurse perspectives from New Zealand. Int J Nurs Stud. 2018;83:18–24. 29684831 10.1016/j.ijnurstu.2018.04.005

[21] Clayton J, Isaacs AN, Ellender I. Perioperative nurses’ experiences of communication in a multicultural operating theatre: A qualitative study. Int J Nurs Stud. 2016;54:7–15. 24680876 10.1016/j.ijnurstu.2014.02.014

[22] Johannesen LM, Hellesø R. Native language nurses and second language nurses document information in patient records differently. Sykepleien forskning (Oslo). 2019:e–76342.

[23] Purnell L. Cross Cultural Communication: Verbal and Non-Verbal Communication, Interpretation and Translation. In: Douglas M, Pacquiao D, Purnell L, editors. Global Applications of Culturally Competent Health Care: Guidelines for Practice. Cham: Springer; 2018.

[24] Baluyot C. Using action research as the basis for transforming a workplace culture so that integration of overseas registered nurses to the nursing home clinical setting be more adaptive, inclusive and mutually supportive. J Compr Nurs Res Care. 2019;4(1).

[25] Astier-Peña MP, Martínez-Bianchi V, Torijano-Casalengua ML, Ares-Blanco S, Bueno-Ortiz JM, Férnandez-García M. The Global Patient Safety Action Plan 2021–2030: Identifying actions for safer primary health care. Aten Primaria. 2021;53:102224. 34961576 10.1016/j.aprim.2021.102224 PMC8721340

[26] Dietl JE, Derksen C, Keller FM, Lippke S. Interdisciplinary and interprofessional communication intervention: How psychological safety fosters communication and increases patient safety. Front Psychol. 2023;14:1164288. 37397302 10.3389/fpsyg.2023.1164288 PMC10310961

[27] Braun V, Clarke V. Thematic Analysis. A Practical Guide: SAGE Publications; 2021.

[28] Deinboll A, Oddvang TK. Hvorfor holdes det fremdeles muntlige rapporter i helseinstitusjoner når helsepersonell har tilgang til elektronisk pasientjournal? en kvalitativ studie. Nordisk tidsskrift helseforskning. 2021;17(2).

[29] Bressan V, Mio M, Palese A. Nursing handovers and patient safety: Findings from an umbrella review. J Adv Nurs. 2020;76(4):927–38. 31815307 10.1111/jan.14288

[30] Burke CS, Salas E, Wilson-Donnelly K, Priest H. How to turn a team of experts into an expert medical team: guidance from the aviation and military communities. Qual Saf Health Care. 2004;13(suppl 1):i96–104. 15465963 10.1136/qshc.2004.009829 PMC1765796

[31] Edmondson AC, Higgins M, Singer S, Weiner J. Understanding Psychological Safety in Health Care and Education Organizations: A Comparative Perspective. Res Hum Dev. 2016;13(1):65–83.

[32] Martins T, Bellagamba D, Simon M, Bucher Andary J, Zúñiga F. Patient safety culture in home care and its related factors and outcomes: a scoping review. BMC Health Serv Res. 2025;25(1):1531. 41299612 10.1186/s12913-025-13732-3 PMC12659234

[33] Althobaiti FM. The effects of leadership on patient safety culture in health care: a systematic review. BMC Nurs. 2026;25(1):125. 41507881 10.1186/s12912-025-04263-7 PMC12882623

[34] Karatuna I, Jönsson S, Muhonen T. Workplace bullying in the nursing profession: A cross-cultural scoping review. Int J Nurs Stud. 2020;111:103628. 32932063 10.1016/j.ijnurstu.2020.103628

[35] Woodhead C, Stoll N, Harwood H, Alexis O, Hatch SL, Bora-White M, et al. They created a team of almost entirely the people who work and are like them: A qualitative study of organisational culture and racialised inequalities among healthcare staff. Sociol Health Illn. 2022;44(2):267–89. 34866199 10.1111/1467-9566.13414 PMC7614856

[36] Rimal RN, Real K. Understanding the Influence of Perceived Norms on Behaviors. Communication theory. 2003;13(2):184–203.

[37] Hovland OJ, Johannessen B. Nursing students develop cultural competence during student exchanges in Tanzania. Sykepleien forskning (Oslo). 2018:e–73782.

[38] Campinha-Bacote J. Delivering patient-centered care in the midst of a cultural conflict: the role of cultural competence. Online J Issues Nurs. 2011;16(2):5. 22088154

[39] Dai X, Chen G-M. Intercultural communication competence: conceptualization and its development in cultural contexts and interactions. 1 ed. Newcastle upon Tyne: Newcastle upon Tyne: Cambridge Scholars Publishing; 2014.

[40] Nembhard IM, Edmondson AC. Psychological Safety: A Foundation for Speaking Up, Collaboration, and Experimentation in Organizations. In: Spreitzer GM, Cameron KS, editors. The Oxford Handbook of Positive Organizational Scholarship. Oxford University Press; 2011. p. 0.

[41] Jiménez P, Winkler B, Dunkl A. Creating a healthy working environment with leadership: the concept of health-promoting leadership. Int J Hum resource Manage. 2017;28(17):2430–48.

[42] Edmonson C, Marshall J, Gogek J. Creating a Healthier Workplace Environment in an Era of Rising Workforce Pressures. Nurs Adm Q. 2021;45(1):52–7. 33259371 10.1097/NAQ.0000000000000448

[43] Tong A, Sainsbury P, Craig J. Consolidated criteria for reporting qualitative research (COREQ): a 32-item checklist for interviews and focus groups. Int J Qual Health Care. 2007;19(6):349–57. 17872937 10.1093/intqhc/mzm042

[44] Tracy SJ. Qualitative Quality: Eight Big-Tent Criteria for Excellent Qualitative Research. Qualitative Inq. 2010;16(10):837–51.

[45] UiA. Research Ethics Committee and Registration of Literature Studies. and REK approved projects and ethical approved projects approved at other institutions, UiA2024 [Available from: https://www.uia.no/english/about-uia/faculty/health-and-sport-sciences/about/fek.html

[46] Bierer BE. Declaration of Helsinki—Revisions for the 21st Century. JAMA. 2025;333(1):18–9. 39425949 10.1001/jama.2024.22281

[47] World Medical Assosiation. Declaration of Helsinki - Ethical prinsiples for medical reasearch involving human subjects 2013. Available from: https://www.wma.net/policies-post/wma-declaration-of-helsinki-ethical-principles-for-medical-research-involving-human-subjects/

