# Supplementary material for: Communication and interaction between native-speaker Norwegian nurses and nurses with Norwegian as a second language in home-based care settings: a qualitative exploratory interpretive study
Source: BMC Nurs. 2026 May 30;25:677. doi: 10.1186/s12912-026-04834-2 (PMC13430762; doi:10.1186/s12912-026-04834-2)
Supplement: Supplementary file 1 — Supplementary Material 1 [file 12912_2026_4834_MOESM1_ESM.docx]

**Interview guide**

Background information

- - Age
  - When and where did you receive your education?
  - How long have you worked in municipal health services?
  - Why did you choose to work in municipal health services?
  - Language: What is your first language? If Norwegian is your second language, how many years have you spoken Norwegian?
  - How many languages and which languages do you speak?

How do you experience the communication with your colleagues?

- - Oral?
  - Written?
  - Nonverbal?
  - Technical/professional terminology
  - Social codes

How do you experience understanding others and being understood?

- What, if any, challenges exist regarding language?
  - Please provide examples if possible

How do different languages and cultural backgrounds impact your work life

- - Please give examples.

Closing

- - Is there anything else you would like to tell us that you feel affects communication between you and your colleagues?

Follow-up prompts

- Can you elaborate on that…?
- What did you think at that moment…?
